# Combination of Common Problem in a Rare Disease: Right Iliac Fossa Pain in a Chronic Myeloid Leukemia Patient

**DOI:** 10.7759/cureus.11523

**Published:** 2020-11-17

**Authors:** Ahmed Faidh Ramzee, Mohammad Sameer, Mohammad Burhan Khan, Syed Muhammad Ali, Ahmad Zarour

**Affiliations:** 1 Surgery, Hamad Medical Corporation, Doha, QAT; 2 Acute Care Surgery, Hamad Medical Corporation, Doha, QAT; 3 Acute Care Surgery, Hamad Medical Corporation, Doho, QAT; 4 Surgery, Weill-Cornell Medical School, Doha, QAT; 5 Acute Care Surgery, Hamad General Hospital, Doha, QAT

**Keywords:** leukemia, appendicitis, typhlitis, abdominal surgery, immunocompromised

## Abstract

Abdominal symptoms in patients with hematological malignancies can occur due to an array of pathologies. Two diagnoses with similar presentation albeit, generally opposite treatment modalities, are typhlitis (inflammation of cecum) and acute appendicitis. Both diagnoses have to be kept in mind in such a patient presenting with right lower quadrant (RLQ) pain. Sagacious clinical judgment along with the aid of radiological imaging may help in differentiating between the two conditions. We present a case of a young male with chronic myeloid leukemia (CML) on imatinib, diagnosed and started on therapy four years earlier, who presented with symptoms of RLQ pain not typical of acute appendicitis. The accurate diagnosis was made with the assistance of ultrasound (US) imaging and prompt surgical therapy was instituted followed by a smooth postoperative recovery.

## Introduction

Acute appendicitis is one of the most common reasons for emergent hospital admissions worldwide with a lifetime risk reaching up to 8% in the general population [1]. However, the incidence of acute appendicitis in patients with leukemia has been reported to be much lower ranging from 0.5% to 1.5% [2]. Leukemia presents a unique dilemma in patients presenting with abdominal pain as a variety of differential diagnoses may be attributed to this condition due in part to the disease process and the treatment itself, ranging from necrotizing enterocolitis, typhlitis, acute appendicitis, acute cholecystitis, and splenic infarction to name a few [3]. The dilemma weighs more in those presenting with right lower quadrant pain as the main differentials in such patients would be typhlitis, enterocolitis, and acute appendicitis requiring polar opposite treatment options, medical and surgical management respectively [4]. Differentiating between these conditions is not straightforward and has been vexing physicians for decades as the signs and symptoms may be nonspecific and diagnosis may be delayed [2]. Good clinical acumen along with appropriate imaging is required to reach the right diagnosis of acute appendicitis as a delay in intervention may lead to life-threatening complications in immunocompromised patients with leukemia [2].

## Case presentation

**Figure 1:**
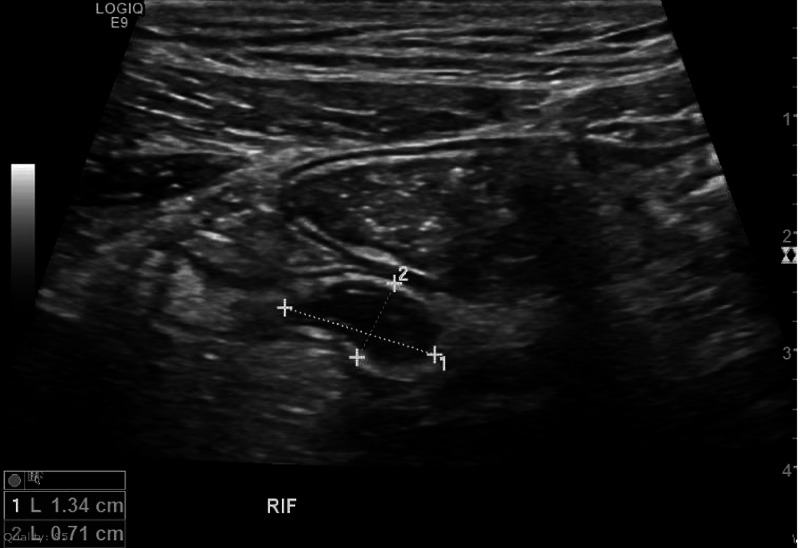
Ultrasound image of the right iliac fossa demonstrating a blind-ending tubular structure distended with fluid measuring 13 mm x 7 mm impressive of obstructed inflamed appendix.

A 28-year-old male diagnosed with chronic myeloid leukemia (CML) in December 2014 and started on imatinib, presented to the ED with two days history of abdominal pain, initially generalized but eventually shifting to the right iliac fossa. This was associated with the passage of loose watery stools although he did not have nausea, vomiting, or loss of appetite. He was afebrile with a heart rate of 80 beats per minute and normal blood pressure of 115/70 mmHg. He was a thin and lean person with a BMI (body mass index) of 22. On examination, he displayed right iliac fossa tenderness with rebound tenderness. His white blood cell count was 6.10 x 103/uL while the absolute neutrophil count was 3.3 x 103/uL, hemoglobin was 15 g/dL, and platelets were 240/mm3; however, C-reactive protein level was mildly elevated, i.e. 39 mg/L (Normal: 10 mg/dL). His Alvarado score was only two showing appendicitis as an unlikely diagnosis. However, his ultrasound (US) examination of the abdomen revealed a 13 mm x 7 mm blind-ending tubular structure distended with fluid at the right iliac fossa impressive of an obstructed inflamed appendix (Figure 1).

He was started on IV antibiotics (cefuroxime and metronidazole) and a laparoscopic appendectomy was performed. Intraoperative findings were remarkable for an obviously inflamed appendix without any perforation or pus in the abdominal cavity. He had a smooth postoperative course during which antibiotics were continued. He was discharged the following day on oral antibiotics. He was seen two weeks later and was doing well. Histopathological examination confirmed the diagnosis of acute appendicitis.

## Discussion

Acute appendicitis is one of the most common surgical emergencies worldwide with an incidence of up to 8% in the general population [1]. However, it seems to be rarer in patients with leukemia where a range of 0.5%-1.5% is reported [2]. CML is a myeloproliferative neoplasm characterized by uncontrolled and dysregulated proliferation of granulocytes with near-normal differentiation occurring as a result of the fusion of two genes: BCR (on chromosome 22) and ABL1 (on chromosome 9) resulting in uncontrolled production of mature and mature granulocytes. Abdominal pathologies complicating leukemia portends a dilemma in terms of an accurate and timely diagnosis. Alterations in the immune defense system as a result of the disease process itself may lead to an increased risk of infectious and inflammatory disease processes [3]. Additionally, patients receiving therapy (chemo/radiation) may be at a higher risk of developing immunosuppression [3]. Notably, the kinase inhibitor; imatinib commonly used in the management of patients with leukemia is known to cause neutropenia in 35%-45% of patients with CML in the chronic phase [3-4]. Our patient was on imatinib at the time of the presentation.

Atypical presentation of acute appendicitis may lead to delay in diagnosis and increases the likelihood of grave complications like perforation, local or generalized peritonitis, and sepsis [2]. The symptoms of appendicitis may be vague and mimic enterocolitis which is a common manifestation of the immunocompromised and neutropenic patients, especially on chemotherapy as was the case with our patient. The other diagnosis that is anatomically related but requires a different modality of treatment is typhlitis [5]. Acute appendicitis usually requires urgent surgical intervention to avoid complications such as perforation, peritonitis, and sepsis [6]. Urgent intervention would be more crucial in immunocompromised patients such as those with leukemia to avoid these devastating complications. This has, however, not been devoid of debate with recommendations evolving over the past decades from medical management to acceptance of the safety and benefits of surgical intervention in leukemic patients [3, 5].

Typhlitis or necrotizing enterocolitis is a condition commonly affecting the cecum in neutropenic patients such as those with hematologic malignancies especially on cytotoxic chemotherapy [7]. The pathogenesis is thought to be explained by damage to the rapidly dividing bowel mucosa caused by chemotherapy eventually leading to bacterial invasion and necrosis of the layers of the bowel [8]. This condition usually presents with fever, abdominal pain [right lower quadrant (RLQ) if terminal ileum or cecum is involved], vomiting, and diarrhea [8-9]. Typhlitis in contrast to acute appendicitis is generally managed with bowel rest, resuscitation, and antibiotics while surgical intervention is recommended only in the presence of complications such as bowel perforation or persistent bleeding [7, 9]. Diagnostic laparoscopy may be indicated in cases where the diagnosis is doubtful as imaging findings are usually nonspecific [9]. Another factor that historically impacted management was the question of the safety of surgery in neutropenic patients. Earlier reports questioned the value of surgery in such patients [10]; however, more recent data advocate early surgical intervention in cases where it is warranted [11]. 

Given the similar clinical presentation of both entities, reaching an accurate surgical diagnosis is pertinent to be able to initiate the appropriate therapy at the earliest. Our patient did not present with the typical symptoms of anorexia, nausea, or vomiting and had a normal white blood cell count with only a slightly elevated C-reactive protein (CRP) level (Alvarado score of 2). The presence of watery stools would add to the confusion; however, we were able to accurately diagnose acute appendicitis with the assistance of an US examination. Ultrasonography is generally regarded as a first-line diagnostic test in acute appendicitis and has shown to have a sensitivity and specificity of 86% and 81% respectively [12]. In typhlitis, the US has reportedly been used with good yield, identifying certain features that may point towards the diagnosis, such as; absent or decreased bowel peristalsis in the RLQ, thickened hypoechoic bowel wall, and markedly thickened echogenic mucosa producing a target or halo sign [13-14]. CT abdomen may reveal more information and help to differentiate between the two diagnoses with more accuracy and is considered the gold standard [15]. It can help demonstrate bowel wall thickening, a dilated cecum or another colonic segment, an inflammatory mass, pericolic inflammation, and pneumatosis intestinalis which would point more in favor of typhlitis [2, 15].

## Conclusions

Physicians should be aware of the complexities abound in patients with hematological malignancies and keep in mind all the possible differential diagnoses in such individuals who present with abdominal pain. Accurate clinical acumen aided by radiological imaging is required to decide on the appropriate path of management. The CT abdomen can delineate many possible pathologies in the right iliac fossa. When in doubt and in patients who may be deteriorating a diagnostic laparoscopy may be warranted.
